# A cross-sectional study of US rural adults’ consumption of fruits and vegetables: do they consume at least five servings daily?

**DOI:** 10.1186/1471-2458-12-280

**Published:** 2012-06-01

**Authors:** M Nawal Lutfiyya, Linda F Chang, Martin S Lipsky

**Affiliations:** 1 Essentia Institute of Rural Health, Division of Research, 502 East 2nd Street, Duluth, MN, 55805, USA; 2 Department of Community and Family Medicine, University of Illinois-Chicago College of Medicine at Rockford, 1601 Parkview Avenue, Rockford, IL, 61107, USA

**Keywords:** Rural health, Fruit and vegetable consumption, Adult nutrition, BRFSS

## Abstract

**Background:**

Rural residents are increasingly identified as being at greater risk for health disparities. These inequities may be related to health behaviors such as adequate fruits and vegetable consumption. There is little national-level population-based research about the prevalence of fruit and vegetable consumption by US rural population adults. The objective of this study was to examine the prevalence differences between US rural and non-rural adults in consuming at least five daily servings of combined fruits and vegetables.

**Methods:**

Cross-sectional analysis of weighted 2009 Behavioral Risk Factor Surveillance Survey (BRFSS) data using bivariate and multivariate techniques. 52,259,789 US adults were identified as consuming at least five daily servings of fruits and vegetables of which 8,983,840 were identified as living in rural locales.

**Results:**

Bivariate analysis revealed that in comparison to non-rural US adults, rural adults were less likely to consume five or more daily servings of fruits and vegetables (OR = 1.161, 95% CI 1.160-1.162). Logistic regression analysis revealed that US rural adults consuming at least five daily servings of fruits and vegetables were more likely to be female, non-Caucasian, married or living with a partner, living in a household without children, living in a household whose annual income was > $35,000, and getting at least moderate physical activity. They were also more likely to have a BMI of <30, have a personal physician, have had a routine medical exam in the past 12 months, self-defined their health as good to excellent and to have deferred medical care because of cost. When comparing the prevalence differences between rural and non-rural US adults within a state, 37 States had a lower prevalence of rural adults consuming at least five daily servings of fruits and vegetables and 11 States a higher prevalence of the same.

**Conclusions:**

This enhanced understanding of fruit and vegetable consumption should prove useful to those seeking to lessen the disparity or inequity between rural and non-rural adults. Additionally, those responsible for health-related planning could benefit from the knowledge of how their state ranks in comparison to others vis-à-vis the consumption of fruits and vegetables by rural adults---a population increasingly being identified as one at risk for health disparities.

## Background

Similar to the US Healthy People 2000 (HP 2000) and HP 2010 objectives, Healthy People 2020 (HP2020) [1] contains nutrition related objectives that recommend Americans increase their consumption of both fruits and vegetables [2]. The HP 2020 objectives for fruit and vegetable consumption echo the 2010 Dietary Guidelines for Americans that recommend an increase in vegetable and fruit intake for all Americans aged 2 years and older [2]. Furthermore, the newly released guidelines emphasize the importance of consuming a variety of vegetables (i.e. dark-green, red, orange vegetables) and beans and peas [2]. Increasing the consumption of fruits and vegetables is deemed an important public health issue since adequate fruit and vegetable consumption may reduce the risk for several major causes of morbidity and mortality in the U.S. including type 2 diabetes [3], heart disease [4,5], stroke [6] and obesity [7]. Moreover, research also suggests that diets rich in fruits and vegetables are associated with a lower incidence of several cancers [8,9], suggesting that dietary choice is also an important cancer prevention measure [10-12].

In addition to reducing the risk of developing many chronic diseases, there is an increasing and compelling body of clinical evidence supporting the benefit of diet and physical activity in not only health maintenance and disease prevention but also for disease treatment, a process referred to as Medical Nutrition Therapy (MNT) [13]. MNT is an essential component in the management and treatment of conditions such as type 2 diabetes, heart disease, hyperlipidemia, stroke and obesity [14-16]. Several widely disseminated clinical practice guidelines advise eating diets high in whole foods such as fruit, vegetables, and whole grains along with limiting animal protein and avoiding high energy low nutrient foods as an important component of disease management.

Another key HP2020 objective is to identify and track segments of the US population experiencing health inequities with the goal of eliminating disparities. Individuals living in rural settings are one population increasingly being identified as at risk for health disparities [1,17-19]. Health inequities or disparities are differences in health between societal strata or groups that are not only avoidable but that are also unnecessary, unfair and unjust [20]. Rural populations in the US experience a disproportionate burden of a number of chronic conditions and many of the major public health problems in rural areas such as obesity, diabetes and tobacco use require or at the very least call for population-level prevention-based interventions.

Hartley [17] urges researchers who have undertaken the examination of the health of rural Americans to explore why rural residency including culture, community and environment, reinforce negative health behaviors. Other researchers [19] aver that examining how and if rural residency affects health behaviors are equally important and that focusing on such may identify rural residency as a fundamental social cause [21] of health inequities.

While researchers have identified differences in fruit and vegetable intake related to race and ethnicity [22-24], age [25,26], socioeconomic factors [22,27,28] and sex [25,29], there is little population-based, national level research examining the consumption of fruits and vegetables by rural populations in the United States. Those studies we found investigating the fruit and vegetable intake of rural populations were limited because they either focused on regional or narrowly defined US populations (e.g., only Hispanic and African American groups or older rural adults) or rural settings outside the US [30-32]

Ultimately focusing on US adults living in rural areas acknowledges the importance of *place* in social epidemiology and public health concerns [33]. Specifically, when assessing health status, health service utilization, health service deficits, adequacy of health care, and health related behaviors---place or geography matters. It has long been held that the places where people live, work and play either protect and/or promote their health and contribute to the health risks they experience [34]. Previous research has indicated that rural residency is an independent risk factor for obesity [35,36], diabetes [37,38], and cardiovascular disease [37] suggesting that dietary habits such as the consumption of fruits and vegetables by rural individuals may differ from their non-rural counterparts.

This study examined the prevalence differences between US rural and non-rural adults in the consumption of at least five daily servings of combined fruits and vegetables. In addition, this study explored what characteristics if any were associated with rural adults consuming five fruits and/or vegetable servings daily. Finally, this study examined by State the prevalence differences between rural and non-rural adult consumption of at least five daily servings of fruits and vegetables.

## Methods

### Data source

Data from the 2009 Behavioral Risk Factor Surveillance Survey (BRFSS), were examined to determine if there were disparities and/or differences between rural and non-rural adults in regard to the daily consumption of at least five servings of combined fruits and vegetables. BRFSS is a cross-sectional, random digit telephone survey that is a collaborative project of the Centers for Disease Control and Prevention (CDC) and all US states and territories. The survey measures several behavioral risk factors in the adult population aged 18 years and older. Its objective is to collect uniform, state-specific data on preventive health practices and risk behaviors linked to chronic diseases, injuries and preventable infectious diseases in the non-institutionalized adult US population.

In this survey, data are collected from a random sample of adults (one per household). All BRFSS data are self-reported responses to mostly forced-choice questions. No additional data are generated to corroborate or substantiate the self-reported responses. As recommended by the CDC, all analyses were performed on weighted data. The weighting provides a stratified representation of the US adult non-institutionalized population that conforms to census data. A more detailed description of the sampling methodology of BRFSS is available elsewhere [39].

### Defining rurality

While there are multiple ways of defining rural for both research as well as policy purposes [40-44], deciding on the specific definition of rural to apply or adopt depends on the purpose of the study, the data used in the analyses, and the appropriate and available taxonomy [42]. Ultimately, there is no perfect definition of rural [42]. In the analyses conducted here, the Metropolitan Statistical Area (MSA) variable included in BRFSS was used to define place of residence as either rural or non-rural. This was the only variable available in the dataset that could be used to define rural or geographical place of residency. As such rural residents were defined as persons living either within an MSA that had no city center or outside an MSA. Non-rural residents included all respondents living in a city center of an MSA, outside the city center of an MSA but inside the county containing the city center, or inside a suburban county of the MSA. The MSA taxonomy was developed by the US Office of Management and Budget and is used by the US Census Bureau and other government agencies for statistical and data analysis purposes [40].

### Dependent variable

The dependent variable for this analysis was consumption of at least 5 daily servings of combined fruits and vegetables. This was a calculated variable derived from survey participant responses to several questions asked by the interviewer administering the survey (see Table 1). Combined fruits and vegetables was chosen as the measure because in the US, fruits such as avocados, tomatoes, corn, peppers, eggplant, squash, and green beans are often misclassified as vegetables when they are in fact fruit. By combining fruits and vegetables we overcome these common misclassifications. Using the measure of a combined five or more servings of fruits and vegetables is consistent with the measurement of fruit and vegetable consumption used in some earlier research [7,45]. Furthermore, low fruit and vegetable consumption has been defined by the World Health Organization panel on diet, nutrition, and prevention of chronic diseases as consuming fewer than five servings of fruits and/or vegetables daily [45].

**Table 1:** Original Survey Question and Response Categories with Re-Coded Response Categories 2009 BRFSS Data

**Analysis Variable**	**Survey Question**	**Original Response Categories**		**Re-coded Response Categories**
**Fruit and Vegetable Consumption**	Not counting juice, how often do you eat fruit?	Calculated variable for consumed five or more servings of fruits or vegetables per day derived from the servings per day variables.	Respondents that reported they never consumed fruits and vegetables or consumed less than 5 servings per day	less than 5 servings per day
	How often do you eat green salad?		Respondents that reported they consumed 5 or more servings of fruits and vegetables per day	5 or more servings of fruits and vegetables per day
	Not counting carrots, potatoes, or salad, how many servings of vegetables do you usually eat? (Example: A serving of vegetables at both lunch and dinner would be two servings.)		Respondents who reported they didn’t know the servings consumed per day, those who refused to answer, and those with missing responses	Missing
**Sex**	Indicate sex of respondent.	Male		Male
		Female		Female
**Race and Ethnicity**	Which one of these groups would you say best represents your race?	Race responses were combined with Hispanic variable to create the second column categories		
		White	White, non-Hispanic	Caucasian
		Black or African American	Black non-Hispanic	African American
		Asian	Asian non-Hispanic	Other/multiracial
		Native Hawaiian or Other Pacific Islander	Native Hawaiian or Other Pacific Islander non-Hispanic	
		American Indian, Alaska Native	American Indian, Alaska Native non-Hispanic	
		Other	Other non-Hispanic	
		Multiracial but preferred race not asked	Multiracial non-Hispanic	
		Don’t know/Not sure, Refused	Don’t know/Not sure, Refused	Missing
	Are you Hispanic or Latino?	Yes	Hispanic	Hispanic
		No	Non-Hispanic	
		Don’t know/Not Sure, Refused	Don’t know/Not Sure, Refused	Missing
**Age Range**	What is your age?	_ _ age in years		18-34 Years
				35-64 Years
				> = 65 Years
**Education**	What is the highest grade or year of school you completed?	Never attended school or only kindergarten		<High School
		Grades 1 through 8 (Elementary)		
		Grades 9 through 11 (Some high school)	
		Grade 12 or GED (High school graduate)		Completed High School
		College 1 year to 3 years (Some college or technical school)	
		College 4 years or more (College graduate)		College Graduate
		Refused, Not asked or Missing		Missing
**Marital Status**	Are you: (marital status)	Married		Married or Living with Partner
		A member of an unmarried couple		
		Divorced		Unmarried and Not Living With a Partner
		Widowed	
		Separated	
		Never married	
		Refused, Not asked or Missing		Missing
**Children in Household**	How many children less than 18 years of age live in your household?	Number of children: _ _ = Number of children		At Least One Child
		None		No Children
		Refused or Missing		Missing
**Household Income**	Is your annual household income from all sources:	Less than $10,000		< $35,000
		Less than $15,000 ($10,000 to less than $15,000)		
		Less than $20,000 ($15,000 to less than $20,000)	
		Less than $25,000 ($20,000 to less than $25,000)	
		Less than $35,000 ($25,000 to less than $35,000)	
		Less than $50,000 ($35,000 to less than $50,000)		> $35,000
		Less than $75,000 ($50,000 to less than $75,000)	
		$75,000 or more	
		Don’t know/Not sure, Refused and Not asked or Missing		Missing
**BMI Categories**	About how much do you weigh without shoes?	BMI calculated using weight and height variables		
		__ _ = weight in pounds	BMI calculated using imperial scale: (weight X 703)/ height in inches²	BMI <25
	About how tall are you without shoes?	_ / _ _ = height in feet / inches		BMI 25- < 30
				BMI > =30
		Don’t know/Not sure, Refused, Not asked or Missing	Don’t know/Not sure, Refused, Not asked or Missing	Missing
**Physical Activity**	Now, thinking about the moderate activities you do in a usual week, do you do moderate activities for at least 10 minutes at a time, such as brisk walking, bicycling, vacuuming, gardening, or anything else that causes some increase in breathing or heart rate?	Calculated variable for adults that have reported participating in either moderate physical activity defined as 30 or more minutes per day for 5 or more days per week, or vigorous activity for 20 or more minutes per day on 3 or more days.	Respondents who reported doing enough moderate or vigorous physical activity to meet the recommendations	Getting at least moderate physical activity
	How many days per week do you do these moderate activities for at least 10 minutes at a time?			
	On days when you do moderate activities for at least 10 minutes at a time, how much total time per day do you spend doing these activities?		Respondents who reported doing insufficient moderate or vigorous physical activity to meet recommendations, or respondents that reported doing no moderate or vigorous physical activity	Inactive
	Now, thinking about the vigorous activities you do in a usual week, do you do vigorous activities for at least 10 minutes at a time, such as running, aerobics, heavy yard work, or anything else that causes large increases in breathing or heart rate?		Respondents who reported they didn’t know whether they did moderate or vigorous physical activity or didn’t know how many days or didn’t know how much time they did the activity, those who refused to answer, and those with missing responses	Missing
	How many days per week do you do these vigorous activities for at least 10 minutes at a time?			
	On days when you do vigorous activities for at least 10 minutes at a time, how much total time per day do you spend doing these activities?			
**Have Health Insurance**	Do you have any kind of health care coverage, including health insurance, prepaid plans such as HMOs, or government plans such as Medicare?	Yes		Yes
		No		No
		Don’t know/Not Sure, Refused		Missing
**Have a Personal Physician**	Do you have one person you think of as your personal doctor or health care provider? (If "No" ask "Is there more than one or is there no person who you think of as your personal doctor or health care provider?".)	Yes, only one		Yes
		More than one		
		No		No
		Don’t know/Not Sure, Refused, Not asked or Missing		Missing
**Timing of Last Routine Medical Check-up**	About how long has it been since you last visited a doctor for a routine checkup? A routine checkup is a general physical exam, not an exam for a specific injury, illness, or condition.	Within past year (anytime less than 12 months ago)		Within the Past 12 Months
		Within past 2 years (1 year but less than 2 years ago)		More than 12 Months Ago
		Within past 5 years (2 years but less than 5 years ago)	
		5 or more years ago	
		Never	
		Don’t know/Not sure or Refused		Missing
**Deferment of Medical Care Because of Cost**	Was there a time in the past 12 months when you needed to see a doctor but could not because of cost?	Yes		Yes
		No		No
		Don’t know/Not sure, Refused		Missing
**Self-Defined Health Status**	Would you say that in general your health is:	Excellent		Good to Excellent
		Very good		
		Good	
		Fair		Fair to Poor
		Poor	
		Don’t know/Not Sure, Refused, Not asked or Missing		Missing
**Residency by Geographic Locale**	Metropolitan Status Code	In the center city of an MSA		Non-rural
		Outside the center city of an MSA but inside the county containing the center city		
		Inside a suburban county of the MSA	
		In an MSA that has no center city		Rural
		Not in an MSA	

### Covariates

Additionally, a number of covariates were included in the analyses. The mix of covariates chosen for inclusion in the analyses included demographic variables associated with social determinants of health, health services variables associated with receipt of or seeking health care, and health status/health condition variables. The demographic covariates were sex, race and ethnicity, age, education, marital status, children in household and household income. The health services covariates were health insurance status, having a personal physician, timing of last routine medical check-up, and deferment of medical care because of cost. The health status/health condition covariates were self-defined health status, body mass index (BMI), and physical activity. A number of these covariates were re-coded from their original formulation for use in this analysis. Table 1 summarizes the original survey questions and response categories with the re-coded response categories. Missing data were not included in the data analysis.

Age and number of children in the household were the only continuous variables re-coded as categorical ones. The variables education, marital status, household income, have a personal physician, timing of last routine medical check-up, and self-defined health status all had multiple categories that were collapsed into fewer categories for analysis. Race and ethnicity, BMI categories, and physical activity were all calculated variables derived from the responses to several survey questions (see Table 1 for greater detail).

Race and ethnicity was calculated from participant responses to two separate survey questions---one regarding race and the other regarding Latino/Hispanic ethnicity. Combining the responses to these two questions allowed for the derivation of the race and ethnicity variable used here. All race/ethnicity categories were computed as mutually exclusive entities. For example all respondents coded as Caucasian chose white as their racial classification, likewise black for African American, etc. If a respondent identified themselves as Hispanic, they were classified by that ethnic category regardless of any additional racial classification.

BMI was calculated from two survey questions, the first asking the respondents height in feet and inches and the second their weight in pounds. The BMI formula BMI = weight in pounds × 703/height in inches^2^ was then used to calculate BMI and code the resultant number into one of three categories: BMI < 25 (neither overweight nor obese), BMI > 25 - < 30 (overweight), and BMI > 30 (obese).

Level of physical activity was calculated by combining other variables assessing physical activity level by: 1) whether or not a person was getting recommended levels of moderate physical activity, and 2) whether or not a person was getting recommended levels of vigorous physical activity. People who reported getting recommended levels of either moderate or vigorous physical activity were coded as getting at least recommended levels of moderate physical activity. Recommended levels of moderate physical activity were defined as moderate-intensity activities such as brisk walking for at least 30 minutes per day, at least five days a week. Respondents getting less than moderate levels of physical activity were coded as inactive.

### Analysis

Bivariate analysis using unadjusted odds ratios and/or contingency table analysis with a chi square test for statistical significance and multivariate logistic regression using weighted data and adjusted odds ratios as the test statistic were performed. The dependent variable for these analyses was rural US adults consuming at least five daily servings of combined fruits and vegetables. Additionally, ArcView version 10.0 (ESRI, Redlands, CA) was used to map the prevalence differences by State between rural and non-rural adults consuming at least five or more daily servings of fruits and/or vegetables. For this calculation and mapping effort the prevalence of rural adults consuming at least five daily servings of fruits and vegetables by state was compared to the prevalence of their non-rural counterparts in the same state. States were color coded on the map to show if the rural in comparison to non-rural adult population of a state had a higher prevalence or a lower/smaller prevalence of consuming at least 5 daily servings of combined fruits and vegetables.

For all statistical analyses, alpha was set at p < 0.05. Statistical Package for Social Scientists (SPSS, IBM, Chicago, IL) version 19.0 was used to complete all statistical analyses performed for this study. Human subject approval was sought and received from Essentia Health’s IRB as well as the University of Illinois, College of Medicine at Rockford’s IRB.

## Results

### Descriptive analysis

For our study, a single year of data for the year 2009 of non-institutionalized US adults (weighted n = 219,479,823) were analyzed. From the 2009 dataset, a weighted 52,259,789 US adults were identified as consuming at least 5 servings of fruits and vegetables daily of which 8,983,840 were identified as living in rural locales. Bivariate analysis revealed that in comparison to US non-rural adults US rural adults were less likely to consume five or more servings of fruits and vegetables (OR = 1.161, 95% CI 1.160-1.162) (not shown on table). Table 2 displays additional comparative data for US non-rural and rural adults regardless of daily consumption of fruits and vegetables. Most notably higher proportions of rural adults when compared to non-rural ones were: Caucasian, older (> 65 years of age), heavier (BMI > 30), less educated (college graduation), poorer (household income < $35,000), married or living with a partner, and without health insurance. Further, a higher proportion of rural vs. non-rural adults: did not have children living at home, had not had a routine medical check-up in the past 12 months, and self-defined their health as fair to poor rather than good to excellent.

**Table 2:** Characteristics of US Adults by Geographic Locale (Rural/Non-Rural) 2009 BRFSS (weighted n = 219,479,823)

**Variables and Factors**		% Rural* (weighted n = 42,365,517)	% Non-rural* (weighted n = 177,114,306)
**Fruit and Vegetable Consumption**	<5 Servings Daily	78.8	75.6
	At Least 5 Servings Daily	21.2	24.4
**Sex**	Male	48.2	48.8
	Female	51.8	51.2
**Race And Ethnicity**	Caucasian	81.3	65.6
	African American	6.2	10.9
	Hispanic	6.6	15.2
	Other	5.8	8.2
**Age Ranges**	18-34 Years	28.4	30.4
	35-64 Years	51.8	53.0
	> = 65 Years	19.8	16.6
**Education**	<High School	11.8	10.4
	Completed High School	63.5	52.7
	College Graduate	24.6	36.9
**Marital Status**	Married Or Living With Partner	66.4	63.9
	Unmarried/Not Living With A Partner	33.6	36.1
**Children In Household**	No Children In Household	60.0	56.0
	At Least 1 Child In Household	40.0	44.0
**Household Income**	<$35,000	43.6	35.1
	> = $35,000	56.4	64.9
**BMI Categories**	BMI < 25	32.8	37.2
	BMI 25- < 30	36.6	36.2
	BMI > =30	30.6	26.6
**Physical Activity**	Getting At Least Moderate Physical Activity	48.9	49.7
	Inactive	51.1	50.3
**Have Health Insurance**	Yes	83.0	85.0
	No	17.0	15.0
**Have A Personal Physician**	Yes	81.9	80.5
	No	18.1	19.5
**Timing Of Last Routine Medical Checkup**	Within Last 12 Months	65.8	68.2
	Longer Than 12 Months Ago	34.2	31.8
**Deferment Of Medical Care Because Of Cost**	Deferred Medical Care Because Of Cost	15.5	14.7
	Did Not Defer Medical Care Because Of Cost	84.5	85.3
**Self-Defined Health Status**	Good To Excellent	81.7	84.7
	Fair To Poor	18.3	15.3

*All cell percentages by row significantly different by z-test measure p < .05.

### Bivariate analysis

Table 3 displays the results of a bivariate analysis for US adults consuming at least five servings of fruits and vegetables daily. As measured by either odds ratio or chi square, all of the covariates in this bivariate analysis yielded a statistically significant relationship with the dependent variable of consuming at least five daily servings of fruits and vegetables. As a result all of the covariates were entered into the multivariate logistic regression model.

**Table 3:** Bivariate Predictor Variables for US Rural Adults Consuming at Least Five Fruit and Vegetable Servings Daily 2009 BRFSS

**Covariates and Factors**			**Unadjusted Odds Ratio (95% CI)**
**Gender** (Male vs. Female)			.964 (.963, .965)
**Children in Household** (No Children In Household vs. At Least 1 Child In Household)			1.141 (1.139,1.142)
**Marital Status** (Married Or Living With Partner vs. Unmarried/ Not Living With A Partner)			1.130 (1.128, 1.131)
**Household Income** (<$35,000 vs. ≥$35,000)			1.297 (1.295, 1.299)
**Health Status** (Good to Excellent vs. Fair to Poor)			.869 (.868, .871)
**Have Health Insurance** (Yes vs. No)			.891 (.890, .893)
**Health Care Provider** (Have HCP vs. Do Not Have HCP)			1.084 (1.082, 1.086)
**Medical Care Deferment** (Deferred Care Because of Cost vs. Did Not Defer Care)			1.059 (1.057, 1.060)
**Routine Check-Up** (Within Past 12 Months vs. Longer Than 12 Months Ago)			.903 (.902, .904)
**Physical Activity** (Getting At Least Moderate Physical Activity vs. Inactive)			.998 (.997, .999)
Contingency Table Percentages*			
**Covariate and Factors**		Rural**	Non-Rural***
**Race/Ethnicity**	Caucasian	81.3%	67.3%
	African American	5.3%	10.3%
	Hispanic	6.9%	13.3%
	Other/Multiracial	6.6%	9.1%
**Age Ranges**	18-34 Years	26.9%	28.3%
	35-64 Years	49.4%	52.7%
	> = 65 Years	23.7%	19.0%
**Education**	<High School	8.6%	8.3%
	Completed High School	58.2%	47.0%
	College Graduate	33.3%	44.7%
**BMI Categories**	BMI < 25	36.8%	42.0%
	BMI 25- < 30	35.9%	35.0%
	BMI > =30	27.3%	23.0%

** 8,983,840.

*** 43,275,949.

* In all instances column proportions differ significantly from each other by chi-square test at the .05 level.

### Logistic regression analysis

Table 4 displays the results of the multivariate analysis preformed. Consumption of at least five daily servings of fruits and vegetables was the dependent variable for the model that included only rural adults. The logistic regression analysis revealed that rural adults whose daily consumption of fruits and vegetables included at least five servings were more likely to be: female rather than male; African American, Hispanic or multiracial/other rather than Caucasian; married or living with a partner rather than single; living in a household without children; living in a household whose annual income is at least $35,000 compared to an income than less than $35,000; and getting at least moderate physical activity rather than being inactive. Rural adults consuming five or more servings of vegetables daily were also more likely to have: a BMI of <25 or a BMI of 25 to <30 rather than > 30; have a personal physician; have had a routine medical exam in the past 12 months; and self-define their health as good to excellent rather than fair to poor. Rural adults consuming at least five daily servings of fruits and vegetables were also more likely to have deferred medical care because of cost.

**Table 4:** Characteristics of US Rural Adults Consuming at Least Five Daily Fruit and Vegetable Servings 2009 BRFSS

**Predictor Variables and Factors**		**Rural Adults Adjusted Odds Ratio** **(95% CI)**
**Sex**	Male	--*
	Female	1.666 (1.663, 1.669)
**Race And Ethnicity**	Caucasian	--*
	African American	1.127 (1.123, 1.131)
	Hispanic	1.474 (1.469, 1.479)
	Other	1.251 (1.246, 1.255)
**Age Ranges**	18-34 Years	.673 (.671, .675)
	35-64 Years	.675 (.673, .676)
	> = 65 Years	--*
**Education**	<High School	.527 (.525, .529)
	Completed High School	.647 (.646, .648)
	College Graduate	--*
**Marital Status**	Married Or Living With Partner	1.071 (1.069, 1.073)
	Unmarried /Not Living With A Partner	--*
**Children In Household**	No Children In Household	1.052 (1.050, 1.054)
	At Least 1 Child In Household	--*
**Household Income**	<$35,000	--*
	> = $35,000	1.111 (1.108, 1.113)
**BMI Categories**	BMI < 25	1.126 (1.124, 1.129)
	BMI 25- < 30	1.066 (1.064, 1.068)
	BMI > =30	--*
**Physical Activity**	Getting At Least Moderate Physical Activity	1.881 (1.878, 1.885)
	Inactive	--*
**Have Health Insurance**	Yes	.962 (.959, .964)
	No	--*
**Have A Personal Physician**	Yes	1.045 (1.042, 1.047)
	No	--*
**Timing Of Last Routine Medical Checkup**	Within Last 12 Months	1.224 (1.222, 1.226)
	Longer Than 12 Months Ago	--*
**Deferment Of Medical Care Because Of Cost**	Deferred Medical Care Because Of Cost	--*
	Did Not Defer Medical Care Because Of Cost	.897 (.895, .899)
**Self-Defined Health Status**	Good To Excellent	1.148 (1.145, 1.151)
	Fair To Poor	--*

--* reference group.

Rural adults consuming at least five daily servings of fruits and vegetables were approximately 33% less likely to be younger (18–34 years or 35–64 years) than older (65 or older). They also were 35.3% to 47.3% less likely of being educated beyond high school (have less than a high school education or being a high school graduate) than being a college graduate.

### Geographic analysis

Table 5 displays prevalence of rural and non-rural adults consuming five or more daily servings of fruits and vegetables by State. Weighted data were used to calculate the prevalences using BRFSS. Also displayed in Table 5 are the standardized percent differences between the prevalences of rural and non-rural adults consuming five or more daily servings of fruits and vegetables by state. The state prevalences for rural adults ranged from a low of 13.88% in Oklahoma to a high of 28.74 % in Vermont. For non-rural adults the prevalences ranged from a low of 14.44% in Oklahoma to a high of 28.27% in Maine.

**Table 5:** Prevalence of Rural and Non-Rural Adults Consuming Five or More Daily Servings of Fruits and Vegetables 2009 BRFSS Data and 2007 USDA Census Data

**State**	**Prevalence Rural Adults**	**Prevalence Non-Rural Adults**	**% Difference Between Rural and Non-Rural Adults**	State National Ranking for Fruit and Vegetable Production*
Alabama	17.01	20.63	−21.27	17
Alaska	19.79	23.76	−20.04	48
Arizona	25.39	23.13	8.90	12
Arkansas	19.01	19.74	−3.87	35
California	22.90	24.28	−6.01	1
Colorado	21.84	22.58	−3.37	26
Connecticut	25.76	27.57	−7.00	37
Delaware	23.45	22.75	2.96	46
Florida	21.64	23.32	−7.77	11
Georgia	21.94	23.58	−7.50	19
Hawaii	25.79	21.40	17.05	7
Idaho	21.55	25.03	−16.19	34
Illinois	22.63	21.65	4.34	24
Indiana	17.17	20.34	−18.42	25
Iowa	17.82	17.96	−0.78	29
Kansas	17.24	18.48	−7.20	41
Kentucky	17.30	21.96	−26.91	14
Louisiana	14.48	17.25	−19.16	38
Maine	25.93	28.27	−9.04	33
Maryland	21.24	26.99	−27.05	32
Massachusetts	19.66	24.27	−23.47	27
Michigan	21.39	22.05	−3.06	5
Minnesota	19.53	22.70	−16.25	22
Mississippi	14.26	19.02	−33.36	28
Missouri	18.14	18.80	−3.64	23
Montana	24.35	25.88	−6.28	40
Nebraska	21.23	19.45	8.35	43
Nevada	23.19	22.48	3.03	49
New Hampshire	28.45	24.71	13.17	42
New Jersey	N/A	24.83	N/A	20
New Mexico	21.34	22.59	−5.86	15
New York	24.50	25.65	−4.69	4
North Carolina	17.34	21.04	−21.37	6
North Dakota	21.75	21.68	0.30	47
Ohio	18.26	20.94	−14.67	9
Oklahoma	13.88	14.44	−4.07	31
Oregon	23.62	25.41	−7.59	8
Pennsylvania	20.34	24.01	−18.04	3
Rhode Island	N/A	25.53	N/A	44
South Carolina	15.33	17.31	−12.88	21
South Dakota	15.71	14.29	9.04	45
Tennessee	19.73	23.26	−17.90	18
Texas	22.70	22.75	−0.22	10
Utah	19.10	23.25	−21.73	30
Vermont	28.74	27.81	3.26	39
Virginia	22.60	26.89	−19.00	16
Washington	22.60	24.90	−10.17	2
West Virginia	15.90	16.18	−1.81	36
Wisconsin	20.04	21.95	−9.53	13
Wyoming	23.14	21.25	8.20	50
U.S.	20.38	23.07	−13.19	

* USDA Census 2007 Data on Number of Farms and Acreage Dedicated to Fruit and Vegetable Production by State were used to compute ranking [46].

**Figure 1:**
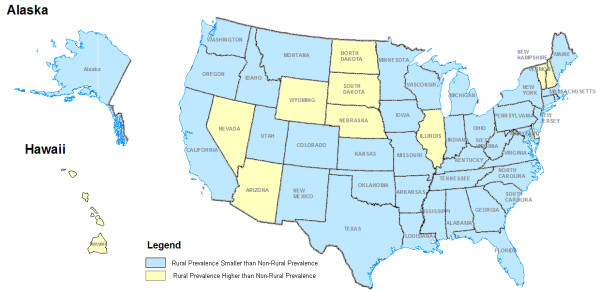
Prevalence of Rural US Adults Consuming at Least Five Daily Servings of Fruits and Vegetables Compared to Non-Rural Adults 2009 BRFSS Data.

The differences in prevalences of fruit and vegetable consumption are presented in Figure 1 with states color coded as having either a higher prevalence (light shade) or a lower prevalence (dark shade) of fruit and vegetable consumption for rural adults in comparison to non-rural adults. Thirty-seven states had lower a prevalence of rural adults consuming at least five daily servings of fruits and vegetables and 11 States a higher prevalence of rural adults consuming at least five daily servings of fruits and vegetables. In two States (New Jersey and Rhode Island) no data on the fruit and vegetable consumption of rural adults were available.

Of the 11 states with a higher prevalence of rural adults consuming at least five daily servings of fruits and vegetables when compared to the non-rural adult population, only one State, Hawaii, was ranked in the top 10 states for fruit and vegetable production. An additional state, Arizona, ranked in the top 20 of fruit and vegetable producing States.

## Discussion

Chronic disease accounts for about 75% of the health care costs in the United Sates and several studies document the benefits of a healthy diet for weight control, and for illnesses such as diabetes, cardiovascular disease and certain types of cancer [3-6,47]. Consuming at least five daily servings of fruits and vegetables are considered an essential part of an overall healthy balanced diet [2]. Our study found that less than 1 in 4 rural US adults consumed five or more servings of fruits and vegetables, a result similar to previous research [48], and a proportion that falls dramatically short of the targets set by HP 2010. Our results also revealed that compared to non-rural adults, a smaller proportion of rural adults reported consuming five servings of combined fruits and vegetables. The findings reported here underscore the continued need for developing targeted interventions that effectively result in healthier dietary choices while addressing possible issues of the availability and accessibility of healthy foods such as fresh fruits and vegetables.

While it may be ironic that rural adults, who live where fruits and vegetables grow, were less likely to consume at least five daily servings it is not necessarily unexpected [48-55]. The importance of community environment as a contributor for individuals adopting a healthy lifestyle, including the availability of low cost health food choices, is increasingly being recognized [50]. Although rural communities produce fruits and vegetables, they typically have fewer stores that offer a wide selection of healthy lower-cost food than non-rural communities [49] and rural residents are more likely to live in a food deserts [56,57]. Since approximately 20% of the US population lives in rural settings [51] strategies aimed at improving access to healthy foods for rural residents could yield significant health benefits. Furthermore, an additional related issue is the ability and/or willingness of rural residents to travel greater distances to food stores where a greater availability of and choices for fruits and vegetables might be found [31].

In addition to environmental access issues, rural residents are typically poorer than their non-rural counterparts and affordability is likely an important contributing factor to fewer rural residents consuming greater amounts of fruits and vegetables. Our results indicate that a higher proportion of rural residents earning less than $35,000 did not consume at least five servings of fruits and vegetables when compared to their non-rural counterparts. Food costs correlate to store type and food tends to be less expensive in larger supermarkets than smaller markets or convenience stores. These higher priced food outlets may be the only local and convenient food source for some rural communities. In addition to a convenience factor, transportation costs may be a barrier to purchasing less expensive healthier food that might be available in a nearby community.

Our findings also reveal several differences in the consumption of fruits and vegetables by characteristics such as gender, age, education, race/ethnicity, physical activity and reported health status. Similar to other studies [22-29], this study found that in rural populations women and those with more education were more likely to consume five or more daily servings of fruits and vegetables. Likewise rural adults over age 65 were more likely to eat at least five servings of fruits and vegetables daily. Data regarding race and ethnicity from previous studies are mixed. In some studies, Caucasians consumed more fruits and vegetables than African Americans while other studies using national data demonstrated the converse [52-55]. Our study found that Caucasians were less likely to consume five servings of fruits and vegetables and that the difference was greater for Caucasians living in rural settings, even though they tended to be better educated and have higher income levels than rural non-Caucasians (combined African Americans, Hispanics, other/Multiracial). The reasons for this difference are not clear and further study to confirm this finding and to understand the reasons why may be helpful in tailoring interventions to improve dietary choices among rural residents.

Those rural residents engaging in at least moderate physical activity and with a lower BMI were also more likely to consume five servings of fruits and vegetables. While physical activity and weight do not directly affect diet choices, our findings add to the body of knowledge that unhealthy lifestyles choices tend to coexist or cluster among individuals [52] just as healthy lifestyle choices do.

Finally, of interest is the distribution of fruit and vegetable consumption by rural and non-rural adults by state. This distribution indicated that in only 11 states did rural adults have a higher prevalence of consuming five or more daily servings of fruits and vegetables than non-rural adults. The reason for this prevalence difference is unclear especially since of those 11 states only one, Hawaii, ranked as a top ten US State for fruit and vegetable production. This finding does suggest the need for further investigation---specifically to answer the question, are there differences between the rural populations in the states where there is a high prevalence of rural adults who are consuming at least five daily servings of fruits and vegetables and those states where such is not occurring? This might provide insight into the role that community environment plays in diets and for what strategies for improving diets might be best suited to a specific rural settings. Also issues such as climate might be more important for rural residents who may be more likely to grow their own food (e.g., vegetables) and could account for some differences among states.

### Limitations

Several potential limitations to this study should be noted. First, the survey is based on telephone derived data and may be skewed because those who could not be reached by phone could not participate in the survey. For example, persons of lower socioeconomic status may have been excluded because of poorer phone access. However, the fact that the vast majority of US residents live in households with telephones minimizes this bias. Furthermore, US cell phone numbers are now included in the pool of phones contacted for the survey. In addition, study strength is the use of a large multi-state database that includes a robust sample of rural residents weighted to reflect the demographics of rural and non-rural US populations.

A second limitation is that the survey used close-ended questions, which limit a responder’s options to fully explain response choices. However, while a different question format may have yielded different results, the survey questions were worded such that the answer choices covered a wide range of response possibilities. A third and related limitation is that the answers are self-reported, which introduces the possibility of recall bias on the part of the survey participants.

Fourthly, the question asking respondents about the number of servings of vegetables is somewhat ambiguous and may have led to an under-reporting of the number of servings of vegetables consumed. For instance no refined measure of consumption was included hence eating vegetables at both lunch and dinner may in actuality constitute more than two servings depending upon the amount of vegetables consumed. Furthermore, the questions did not specifically address vegetables in foods such as stews or soups. However, there is no reason to suspect that there would be reporting differences between rural and non-rural populations suggesting the data can yield meaningful comparisons.

A fifth potential bias resulted from the languages of the survey – English and Spanish. Individuals who did not speak English or Spanish were excluded from this survey. Not all US residents speak the two languages of this survey as a result those adults from other cultures who do not speak either English or Spanish and who have vegetable rich (e.g., Chinese) or fruit and vegetable rich (e.g., Mediterranean) diets may have been excluded and as a result the aggregated data may not be representative of actual consumption by all adults who are residing in the United States.

Finally, in the US, no standard definition exists for rural. In the BRFSS data used in this study, MSA was the only possible definition for rural. The main weakness of the MSA definition is that it does not differentiate well between nonmetropolitan or rural counties. The strengths, however, of the MSA definition are that it is stable over time and it is familiar to policy makers.

## Conclusion

In conclusion, most Americans do not eat the recommended number of servings of fruits and vegetables. However, rural residents appear at greater risk for not making healthy dietary choices. Successfully improving the dietary patterns of Americans will need to incorporate the environmental context in which people live. The enhanced understanding of fruit and vegetable consumption provided from the research reported on here should prove useful to those seeking to lessen the disparity or inequity between rural and non-rural adults in regard to adequate fruit and vegetable consumption. Our findings should be helpful for public health practitioners interested in developing population-level prevention-focused interventions aimed at improving the diets and health of rural Americans and in understanding the importance place plays in the creation of health inequities and the need for developing targeted public health interventions.

## Misc

M. Nawal Lutfiyya; Linda F. Chang; Martin S. Lipsky contributed equally to this work

An earlier draft of this paper was presented to the annual meeting of the National Rural Health Association, 3–6 May 2011, Austin Texas

## Competing interests

The authors declare that they have no competing interests.

## Authors’ contributions

MNL, LFC, MSL all made substantial contributions to conception and design of the manuscript. MNL was responsible for the acquisition of the data used and the analysis of data. MNL, LFC, MSL were equally involved in interpreting the data, drafting the manuscript and revising it critically for important intellectual content. MNL, LFC, MSL have given final approval of the version to be published. All authors read and approved the final manuscript.

## Pre-publication history

The pre-publication history for this paper can be accessed here:

http://www.biomedcentral.com/1471-2458/12/280/prepub
